# Valorization of Amaranth (*Amaranthus cruentus*) Grain Extracts for the Development of Alginate-Based Active Films

**DOI:** 10.3390/molecules27185798

**Published:** 2022-09-07

**Authors:** Laylla Marques Coelho, Carla Faria, Daniel Madalena, Zlatina Genisheva, Joana T. Martins, António A. Vicente, Ana C. Pinheiro

**Affiliations:** 1 CEB—Centre of Biological Engineering, University of Minho, 4710-057 Braga, Portugal; 2 LABBELS—Associate Laboratory, Braga/Guimarães, Portugal

**Keywords:** active films, bio-based packaging, physical properties, antioxidant activity, nutraceutical

## Abstract

This research work investigates the development of alginate-based films incorporating phenolic compounds extracted from *Amaranthus cruentus* grain using different solvents. Alginate, glycerol, and amaranth grain phenolic compounds at various concentrations were used to produce the films. An experimental Central Composite Rotatable Design (CCRD) was used to evaluate the effect of these variables on different film’s properties, i.e., water vapor permeability, hydrophobicity, moisture content, solubility, thermal, mechanical, and optical properties. This study demonstrated that high phenolic compound content and antioxidant capacity were obtained from amaranth grain using ethanol as the extraction solvent. Alginate films incorporating amaranth phenolic compounds were successfully manufactured, and this study can be used to tailor the formulation of alginate films containing amaranth phenolic compounds, depending on their final food application. For example, less flexible but more resistant and water-soluble films can be produced by increasing the alginate concentration, which was confirmed by a Principal Component Analysis (PCA) and Partial Least Squares (PLS) analysis. This study showed that active alginate films with amaranth phenolic compounds can be tailored to be used as food packaging material with potential antioxidant activity.

## 1. Introduction

Plastic films (e.g., polyvinyl chloride and polypropylene) present various deleterious effects to the environment, namely reduction of soil fertility and permeability, which decreases crop yields [1]. To overcome these issues, natural macromolecules and biodegradable materials like polysaccharides can be used as plastic substitutes to produce films. Alginate, a polysaccharide extracted mostly from brown algae cell walls, is composed of guluronic acid and mannuronic acid. Alginate is biodegradable, non-toxic, and has excellent film-forming properties [2].

The development of active packaging materials (e.g., presenting antioxidant activity) is very useful to extend food shelf life, improve food safety or its sensory properties. Most of the recent research studies aim to directly incorporate active compounds into the polymeric packaging, while maintaining or improving the barrier and mechanical properties of the film material. However, the use of synthetic antioxidants such as propyl gallate, butylated hydroxyanisole (BHA), and butylated hydroxytoluene (BHT) in food can cause many adverse side effects [3]. Consequently, there is a growing interest in finding natural antioxidant substances capable of eliminating free radicals and hindering oxidation rancidity and delaying food spoilage. Phenolic compounds are plants’ secondary metabolites presenting a phenol ring (phenolic acids, phenolic alcohols) or various ring aromatic compounds with one or more hydroxyl groups (i.e., polyphenols). In-vivo and in-vitro experiments have revealed that most phenolic compounds, especially polyphenols, have many different bioactivities such as antioxidation, digestive enzyme inhibition, and anti-inflammation properties [4]. Natural compounds with antioxidant properties can be used in food packaging as they may be biologically degradable and are commonly considered safe [5]. For example, the addition of antioxidants such as α-tocopherol or citric acid to edible starch-chitosan films enhanced their antioxidant capacity and barrier properties [6].

Amaranth (*Amaranthus cruentus*) grain is a pseudocereal presenting great potential to prevent malnutrition especially in the low-income food-deficient countries. Additionally, amaranth grains are gluten-free and a rich bioactive compounds’ source. Moreover, the lipid fraction of grains include various compounds (e.g., tocopherols, squalene, and sterols), which present anticancer, antithrombotic, antidepressant, hypocholesterolemic, and antidiarrheal properties [7]. Amaranth grains contain phenolic compounds (e.g., flavonoids) and a relatively high antioxidant capacity, where total phenolic acids in several amaranth ecotypes range from 16.8 to 40.1 mg/100 g (dry weight) [8]. Nevertheless, the phytochemical composition depends on multiple variables during growing conditions [9].

Amaranth flour-based films have already been described [10]. However, phenolic compounds extraction from amaranth grain and their incorporation on alginate-based films has not yet been explored. Therefore, this work could provide an alternative for the food industry, since amaranth biological activities could enhance edible films’ properties. Furthermore, amaranth grain phenolics’ incorporation in edible films could be a route to increase phenolic stability, antioxidant efficiency, and foods’ shelf life. In this context, alginate films incorporating phenolic compounds extracted from amaranth grains were developed, characterized, and their formulation optimized, depending on the application.

## 2. Results and Discussion 

### 2.1. Antioxidant Activity and Total Phenolic Content of Amaranth Grain Extracts

The total phenolic content in the methanolic extract (3.9 ± 0.12 mg GAE·g^−1^ sample) and ethanolic extract (3.5 ± 0.09 mg GAE·g^−1^ sample) was significantly higher (*p* < 0.05) than in aqueous extracts (2.4 ± 0.07 mg GAE·g^−1^ sample) as can be seen in Table 1. Also, the antioxidant activity of the methanolic extract (0.24 ± 0.01 mg ACE·g^−1^ sample) and ethanolic extract (0.21 ± 0.01 mg ACE·g^−1^ sample) was higher (*p* < 0.05) than aqueous extract (0.12 ± 0.02 mg ACE·g^−1^ sample) (Table 1).

These results could be explained by the enhanced solvation of antioxidant compounds present in amaranth grain due to interactions (hydrogen bonds) between the polar sites of the antioxidant compounds molecules and the solvent [11]. Methanol was more efficient in the extraction of antioxidant compounds than ethanol (though they present very similar polarities). This could be due to the higher solvation of antioxidant molecules provided by methanol, possibly due to the presence of the methyl radical [11]. Moreover, the extraction of the antioxidant compounds from amaranth grain could be influenced by changes of solvents’ polarity (decreased in this order: water > methanol > ethanol), which alter its ability to dissolve antioxidant compounds. Usually, phenolic compounds are more soluble in organic solvents, which are less polar than water [12]. Pazinatto, et al. [13] observed a similar behavior for amaranth. Different solvent polarities cause differences not only in the extracted phenolic compounds, but also in the antioxidant capacity found in the extracts [14].

### 2.2. Identification of Amaranth Phenolic Compounds by UHPLC

The total phenolic content results showed no significant differences (*p* > 0.05) between ethanol (3.5 ± 0.09 mg GAE·g^−1^ sample) and methanol extracts (3.9 ± 0.12 mg GAE·g^−1^ sample), according to Tukey’s multiple comparisons test. Therefore, the ethanol extract was chosen to perform the analysis of phenolic compounds, since methanol is considered a more toxic solvent than ethanol for food applications [15].

The phenolic compounds identified in the mixture extracted from amaranth grains are presented in Table 2. 

The principal antioxidant compounds were detected in ethanol *A. cruentus* extracts, in accordance with other reported results [16]. Moreover, Pedersen, et al. [17] detected three main polyphenols (i.e., rutin, isoquercitin, and resveratrol) in amaranth extracts, and rutin presented a higher concentration than isoquercitin and resveratrol. The other amaranth compounds identified by Pedersen, et al. [17] had similar concentration values to our results. Polyphenolic compounds such as ferulic acid (120–620 mg·kg^−1^), vanillic acid (15.5–69.5 mg·kg^−1^), benzoic acids (4.7–136 mg·kg^−1^), caffeic acid (6.41–6.61 mg·kg^−1^), and *p*-coumaric acid (1.2–17.4 mg·kg^−1^) were detected in amaranth according to other authors [8]. Also, flavonoids have been detected in amaranth grain, for instance, quercetin (214–843 mg·kg^−1^), kaempferol (22.4–59.7 mg·kg^−1^), isorhamnetin (42–600 mg·kg^−1^), and rutin (7–592 mg·kg^−1^) [18]. Rutin (quercetin-3-O-rutinoside) and quercetin (rutin precursor) are flavonoids found in amaranth, which present important antioxidant properties, inhibit the oxidation of high-density lipoprotein cholesterol, and could prevent different types of cancer [7].

### 2.3. Film Surface Morphology

**Figure 1:**
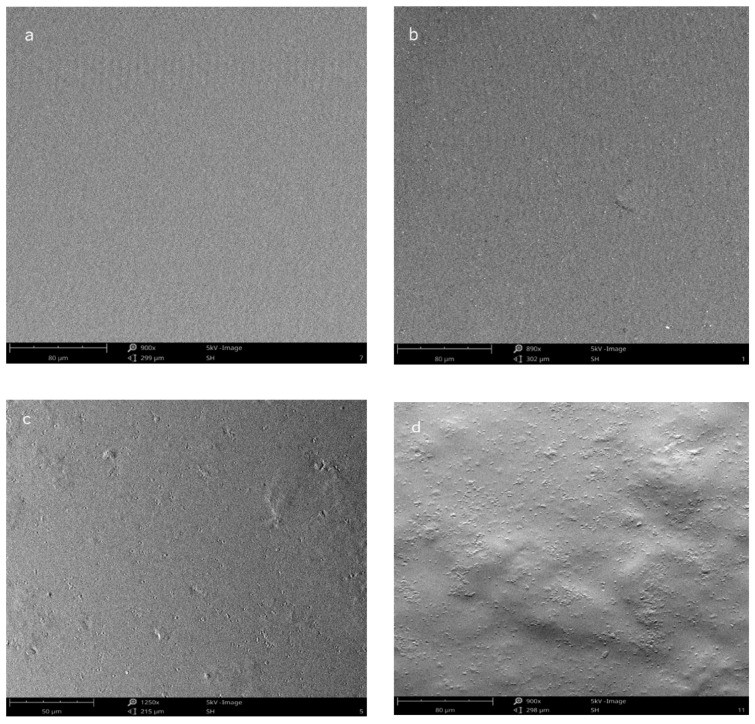
SEM photos of the surface of alginate films with (**a**) 0% (sample 7), (**b**) 0.30% (sample 1), (**c**) 1.00% (sample 5), and (**d**) 2.0% (sample 8) of amaranth phenolic compounds (film samples composition is presented in Section 3).

SEM micrographs of the films containing different amaranth phenolic compounds’ concentrations are shown in Figure 1. Sample 7 surface (with no phenolic compounds added) is very smooth and continuous (Figure 1a). Samples 1 and 5 (films with 0.30% and 1.00% of phenolic compounds’, respectively) showed a homogeneous film surface with some roughness (Figure 1b,c, respectively). On the other hand, film surface became less smooth as the concentration of phenolic compounds increased. Some phenolic compounds aggregates were found in sample 8 (2.00% of phenolic compounds) (Figure 1d). These results were similar to other results reported in the literature, where increasing extract content within the film matrix reduces film surface smoothness [19,20]. 

### 2.4. Water Angle Contact, Solubility, Moisture Content (MC), Water Vapor Permeability (WVP) Measurements of Films

Film surface hydrophobicity is a key property of packaging materials, being assessed by measuring the water contact angle formed between the film surface and a water droplet placed in its surface. Usually, water contact angle is less than 65° when the film surface is hydrophilic [21]. 

Table 3 shows the water contact angles of alginate films with different amaranth phenolic compounds content. The water contact angle of all the film samples did not exceed 65°, indicating a hydrophilic surface. In particular, samples 2, 4, 9, 10, and 11 presented water contact values of 0° because it was not possible to measure the contact angle after 180 s of the droplet placement in the film surface. In fact, the water droplet spread out on these films’ surface, which means that these samples are the ones with low hydrophobic character since the increase of the contact angle amplitude is directly proportional to the increase in hydrophobicity of the film surface. The high hydrophilicity of films’ surface can be explained by the hydrophilic nature of alginate and glycerol [22].

Comparing film samples 1 and 2, which have the same phenolic compounds’ concentration, it is noticeable that sample 2 is more hydrophilic since its contact angle value is lower than sample 1. This situation is also reflected when comparing sample 3 to sample 4. The differences observed between samples 1 and 2 (and samples 3 and 4) could be due to the increase of alginate concentration (0.86 to 1.39%) and simultaneously, the reduction of glycerol content (0.64 to 0.11%) in the films. Glycerol interacts with alginate polymeric chains, reducing the number of free hydrophilic groups of alginate that can interact with water, contributing to an increase of film surface hydrophobicity. However, sample 6 did not follow this tendency despite the absence of glycerol, which is probably related to the influence of hydrophobic phenolic compounds concentration (1% in the film). In fact, sample 3, which has high glycerol and phenolic compounds concentrations (0.64% and 1.7%, respectively), presented the most hydrophobic character (i.e., the highest contact angle value) compared to the other samples. This outcome proved the effect of both glycerol and phenolic compounds in the hydrophobicity of the films.

Film solubility in water provides an insight into the film’s water resistance when applied on water-rich foods and consequently, film biodegradability. Table 3 shows the solubility and *MC* values obtained for alginate films incorporating amaranth phenolic compounds. An increase of phenolic compounds’ concentration on samples 7, 8, and 9–11 (i.e., fixed glycerol and alginate concentrations) led to a significant increase (*p* < 0.05) of *MC* values. However, film solubility of samples 8–11 significantly decreased (*p* < 0.05) with the addition of phenolic compounds. This could be explained by phenolic compounds’ capacity to decreased films’ hydrophilicity. Other authors reported a similar behavior when extracts of purple onion peel were added to alginate-based films [23]. The authors attributed the decrease in films’ solubility to polyphenols interaction with polymeric chains, which decrease the number of accessible hydroxyl groups to water molecules.

Film’s *WVP* is a critical factor to understand the moisture exchanges between the coated product and the surrounding environment. These exchanges depend on many factors, such as film integrity, hydrophilic-hydrophobic ratio, crystalline-amorphous ratio, and polymeric chain mobility [24]. As one of the major purposes of bio-based films is to block moisture transfer between the food and the surrounding atmosphere, the *WVP* should be as low as possible. It can be seen that the *WVP* values of the samples (Table 3) are within the average value range for alginate films [25]. According to Jouki, et al. [26], high-crystalline polymers, such as alginate, are generally less permeable due to their ordered structure and the mass transfer of a gas in a semi-crystalline polymer is mainly a function of the amorphous phase. The higher *WVP* values were obtained for samples 3, 5, and 6. Samples 3 and 5 had high glycerol concentrations. The increase of glycerol concentration increases the free volume and chain movement, reducing the rigidity and increasing the molecular mobility of films, thus allowing a higher water vapor diffusion through their structure [27]. Moreover, the increase of amaranth extract content from 0.30% (samples 1 and 2) to 1.70% (samples 3 and 4), possibly increased the free volume (less compact film structure), and consequently, water vapor molecules diffused more easily within the film matrix, causing the increase of *WVP* values of the alginate films [28]. Likewise, other authors [29] reported an increase in *WVP* of alginate films as a result of the increasing incorporation of star anise extract/hydroxypropyl-β-cyclodextrin complex, which caused an increase of free volume.

### 2.5. Thermal Stability-Thermogravimetric Analysis (TGA) and Differential Scanning Calorimetry (DSC)

Edible films’ application in the food industry needs to consider films’ thermal stability characteristics as they may be subjected to various thermal processes. The most used techniques to study biopolymers’ thermal stability are DSC and TGA. DSC is a conventional method of thermal analysis widely used to characterize the phase transition, and it is used to measure the melting enthalpy (Δ*H_m_*), associated with film crystallinity, and the glass transition temperature (*T_g_*), associated with the system mobility and defined as a physical change from the glassy to the rubbery state in amorphous materials promoted by heat [30]. *T_g_* values of biodegradable films help to choose the best storage conditions, and it is expected that *WVP* of the films will be higher at temperatures higher than the *T_g_* value, where polymer chains are in greatest motion. TGA and DSC results are shown in Table 3.

Thermal analysis showed that films are stable up to 60 °C for all formulations (results not shown). The first stage (60–120 °C) can be attributed to water evaporation and chemisorbed water through hydrogen bonding [31]. The second stage (170–230 °C) is frequently ascribed to glycerol existence [27]. The third stage (230–330 °C) and also, the highest peak in the DTG curve, is linked to polysaccharide breakdown [32]. Regarding the presence of plasticizing agents, typically, glycerol has a great effect on *T_g_* value [27]. It is expected that films with higher glycerol concentration will have a lower *T_g_* value, once the increase of glycerol concentration leads to an increase of the free volume and mobility of molecules, changing the physical structure of the alginate films, which results in the decrease of *T_g_* values. However, this effect was not clear in our study. On the other hand, the Δ*H_m_* and *T_g_* values changed with different phenolic compounds concentrations. For instance, comparing Δ*H_m_* and *T_g_* values of samples 1 and 3, which have the same alginate and glycerol concentration but different phenolic compounds concentration, it can be observed that Δ*H_m_* and *T_g_* values of sample 3 were lower than sample 1 (*p* < 0.05) because it presented higher phenolic compounds amount. The same behavior was observed (*p* < 0.05) between samples 2 and 4, and between samples 7, 9–11, and 8 (equal alginate and glycerol concentration and increasing phenolic compound’ concentrations). The lower Δ*H_m_* values are possibly explained by the decrease of crystallinity of the alginate films due to phenolic compounds addition [33]. 

TGA also allows to obtain the onset temperature (*T_onset_*) (i.e., start-up decomposition temperature) and the *TGA_p_* (the temperature at which the film decomposition reaches maximum rate) (Table 3). Observing *T_onset_* and *TGA_p_* values obtained for the film samples, the lowest values were obtained for sample 6, which does not present glycerol in its formulation. Although it is not very clear in this work, it was expected that the *T_onset_* and *TGA_p_* values would be higher for samples presenting higher glycerol concentrations, due to glycerol depolymerization and pyrolytic decomposition, which only occurs at approximately 250 °C [34].

### 2.6. Mechanical Properties

**Figure 2:**
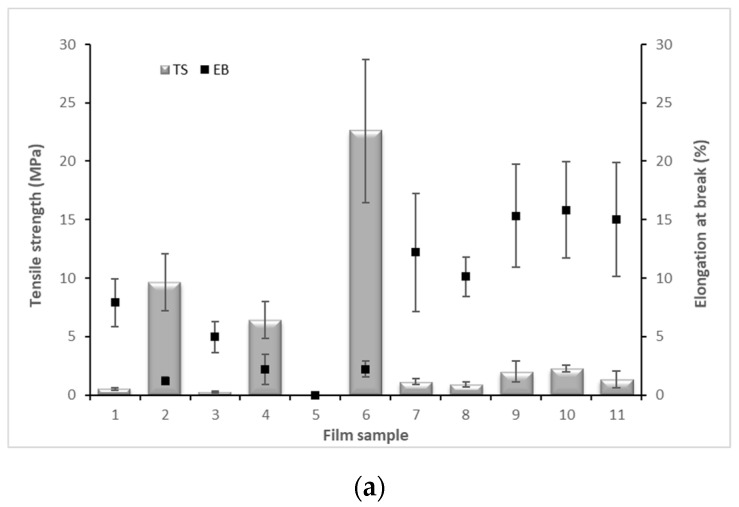

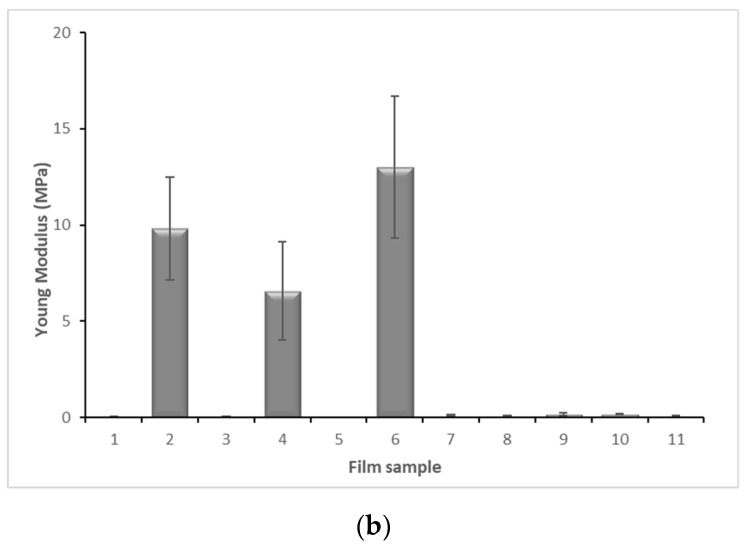
Tensile strength (*TS*), elongation at break (*EB*) (**a**), and Young’s modulus (*YM*) (**b**) of alginate films incorporating amaranth phenolic compounds.

Mechanical properties including elongation at break (*EB*), tensile strength (*TS*), and Young’s modulus (*YM*), reflect films’ flexibility, rigidity, and ability to protect food integrity and durability. The results of mechanical properties of each film sample are presented in Figure 2. 

Sample 5 was not evaluated because it was not possible to handle this sample properly due to the high glycerol amount. On the other hand, sample 6 was the stiffest one, presenting high *YM* values, since it does not contain glycerol (Figure 2). Therefore, these two film samples are not suitable for coating food products because sample 5 is too elastic and difficult to work with, and sample 6 is a very rigid and brittle film that does not have the elasticity to coat a food product.

The *EB* value of sample 7 (0% phenolic compounds and 0.37% glycerol) was 12.19 ± 5.06% and *TS* value was 1.12 ± 0.25 MPa. The *EB* value of the films with 1.00% of phenolic compounds and without glycerol (sample 6) was 2.20 ± 0.69% and *TS* value was 22.58 ± 6.10 MPa. Comparing these samples, it is deduced that the ratio between glycerol concentration and phenolic compounds is involved in the increased flexibility and maximum stress that the film can withstand. Furthermore, comparing samples 1 and 2 that have the same phenolic compounds’ concentration, with glycerol concentration being higher in sample 1, the *EB* value increased when glycerol amount increased, while *YM* and *TS* values decreased. The same behavior was observed when comparing samples 3 and 4. This is a result of the increasing glycerol concentration, which increases polymer chains mobility and creates more extensible films [35]. In fact, plasticizers, such glycerol, are known to interfere with polysaccharides’ chains, decreasing intermolecular forces, softening the rigidity of the films’ structure, increasing polymer mobility, and thus, decreasing *TS* and increasing *EB*. These results are in agreement with the ones reported in the literature that show an increase of *EB* and a decrease of *TS* values with the presence and increasing concentrations of a plasticizer [27].

However, increasing amaranth phenolic compounds content to 2% (sample 8) led to a significant decrease in *EB* (*p* < 0.05) and *TS* values of films compared to samples 9–11 (1% phenolic compounds’ content). These results are in agreement to other authors [36] who reported a decrease of *EB* and *TS* of alginate films when the gallnut extract content was above 25%. According to these authors, the decrease of flexibility and stiffness of alginate/gallnut extract films was attributed to the extract agglomeration and thus, a heterogeneous film matrix was formed.

### 2.7. Optical Properties-Color and Opacity of the Films

Packaging film transparency and color are valuable features because they influence consumer choice and food product quality. The internal and surface film microstructure plays an essential part in film optical properties. 

Overall, alginate-based films showed low opacity (*OP*) values (Table 4) and thus, good transparency, regardless of the glycerol, alginate, and phenolic compounds concentrations used in film composition. In fact, through visual observation, all films have a clear and smooth appearance despite slight differences in *OP* values. Usually, higher thickness values increase *OP* values because it is more difficult to pass light through the film material. Although thickness values did not change significantly (*p* > 0.05) with the different films’ formulations, *OP* values seemed to depend on the films’ composition. It was possible to detect an increase of *OP* values of samples 2 and 4 due to a decrease of glycerol concentration compared to samples 1 and 3, respectively. Moreover, sample 5 (containing the highest percentage of glycerol) presented the lowest *OP* value (*p* < 0.05) comparing to the other samples. *OP* is associated to particles dispersion in the film matrix, once they can block light, causing more light scattering within films [37]. These results are consistent with other authors’ work, who showed that potato flour films are more transparent at higher glycerol content [38]. 

Also, the increase of amaranth extract concentration increased the *OP* values as can be seen in Table 4 for samples 7 to 11 (*p* < 0.05). This might be due to the interaction of phenolic compounds with alginate and glycerol in the film matrix. A similar report indicated that increasing onion peel extract content on sodium alginate/carboxymethyl cellulose/gluten-based films increased *OP* values [20].

Regarding color parameters, Table 4 shows the L*, a*, and b* values. The brightness parameter (L*) provides a black to white scale (0–100), a* is the coordinate for redness and greenness and b* is the coordinate for yellowish coloration. The color parameters L*, a*, and b* revealed the effect of alginate, glycerol, and phenolic compounds on films’ color. The increase in glycerol concentration (samples 1 to 2 and samples 3 to 4) led to a decrease in L* and a* values, indicating a decrease of the lightness and an increase of the greenness of the film. The presence of phenolic compounds also has an influence on the color parameters. According to Corrales, et al. [39], the presence of phenolic acids and flavonoids due to the addition of grape seed extracts in pea starch films led to brownish-orange film color formation (L* and a* values decreased and b* values increased). 

Optical properties’ results indicated that the alginate-based films incorporating amaranth extracts could restrict the passage of light through the film and increase the potential to avoid degradation of light-sensitive food compounds.

### 2.8. Optimization of Alginate-Based Films’ Formulation

A Central Composite Rotatable Design (CCRD) was performed to optimize the different formulations of alginate-based films. The results of the CCRD are expressed in Table 5.

It is possible to observe from the results of Table 5 that not all dependent variables were appropriately modulated by the CCRD. For instance, the samples’ *WVP*, *CA*, *T_g_*, and *TGA_p_* variables showed a high regression *p-value* (*p-value* > 0.05), which indicates that the model was not a good fit to the experimental data and most of the response variability was not explained by the models (i.e., it is not statistically significant). Furthermore, *F_calc_* < *F_tab_* indicated that the studied independent variables did not have a significant effect on the samples’ *WVP*, *CA*, *T_g_*, and *TGA_p_* variables. On the other hand, the samples’ *Sol*, *MC*, *OP*, *YM*, *TS* and *EB* models were statistically significant (*p-value* < 0.05), where *F_calc_* > *F_tab_* (i.e., significant effect on the variables’ response). These findings showed that the models may be used for prediction within the evaluated experimental range. Thus, to further understand the relationship between the different films’ formulations and the dependent variables, a multivariate analysis was conducted using the following dependent variables: *Sol*, *MC*, *OP*, *YM*, *TS*, and *EB*. 

### 2.9. Multivariate Analysis Using Partial Least Squares (PLS), Principal Component Analysis (PCA), k-Means Cluster Analysis and Pearson’s Correlation Matrices

**Figure 3:**
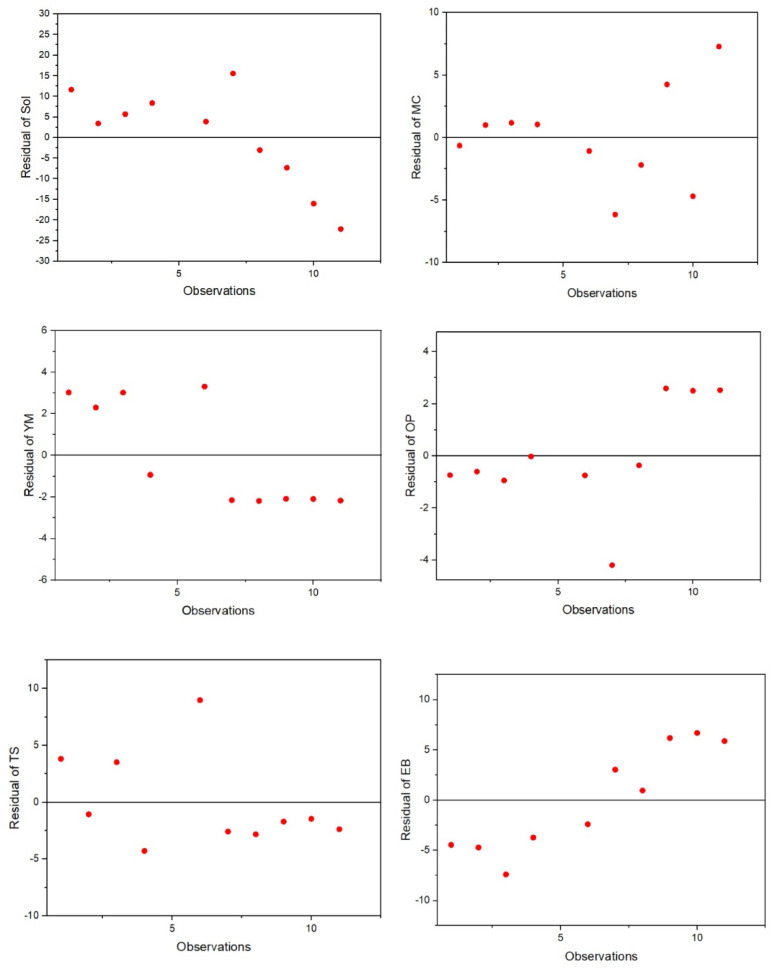
PLS regression residuals’ distribution for the selected dependent variables (*Sol*, solubility; *MC*, moisture content; *YM*, Young Modulus; *OP*, opacity; *TS*, tensile strength; *EB*, elongation at break).

Multivariate techniques (i.e., PLS and PCA) were used to analyze the relationship between films’ parameters and their composition in order to better assess and statistically explain the different behaviors observed in the studied films. The PLS analysis determined that the samples’ alginate content is responsible for the variability in the samples’ *Sol*, *MC*, *OP*, *YM*, *TS*, and *EB*, since it presents a variable importance of projections (VIP) score of 1.41 (VIP > 0.8), while their phenolic compounds’ (*PC*) content did not significantly explain the variability of the dependent variables since it presents a VIP score of 0.00023 (VIP < 0.8). The extracted principal component explains 46.17% of the dependent variables’ variability, which indicates that the PLS regression was an appropriate method to correlate both dependent and independent variables. The PLS quality can be further assessed by analyzing the PLS regression residuals’ distribution. In this case, they presented a random normal distribution around 0 (Figure 3), which indicates that the PLS has a good quality, with the exception of the *EB*, which shows a third degree order tendency, suggesting that a third order polynomial could be used to model this variable. 

**Figure 4:**
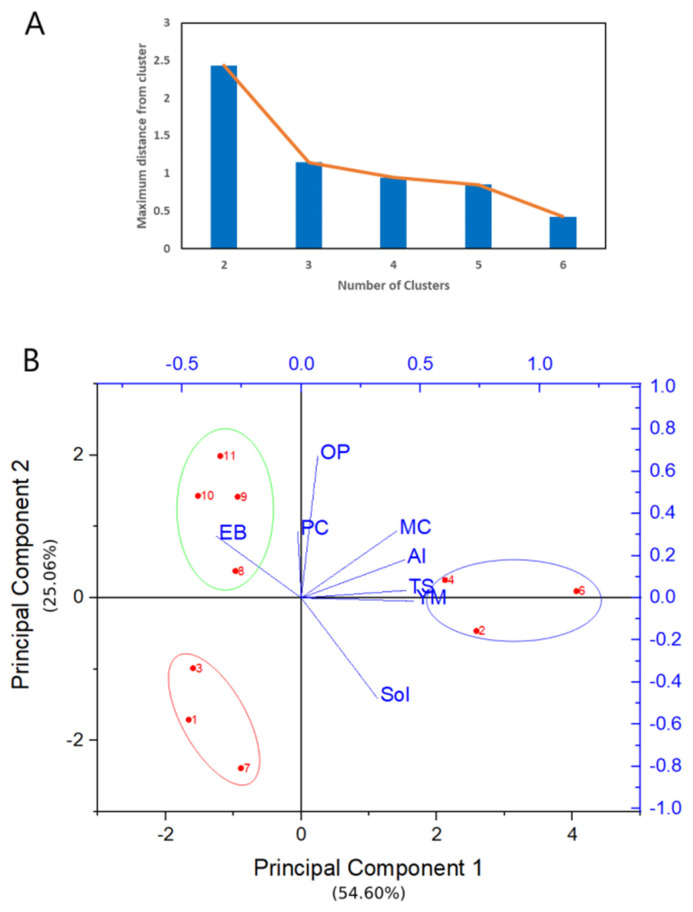
Relationship between the maximum distance to a cluster and the number of clusters during the k-means cluster analysis (**A**) and the PCA biplot between the first two principal components (**B**) where dots represent the scores plot and lines represent the loadings plot (*Al*, alginate; *PC*, phenolic compounds; *Sol*, solubility; *MC*, moisture content; *YM*, Young Modulus; *OP*, opacity; *TS*, tensile strength; *EB*, elongation at break).

A PCA analysis (i.e., scores and loadings plot) was conducted and complemented with a k-means cluster analysis to group the samples based on their similarities. The results are represented in Figure 4. 

From PCA results it is possible to observe that the principal component 1 (*PC*1) explains 54.60% of the total data variability and the principal component 2 (*PC*2) explains 25.06% of the total data variability (Figure 4B). This implies that differences observed in the scores related to *PC*1 are more relevant when compared to differences observed in *PC*2. Furthermore, it is also possible to observe that the variations in *YM*, *MC*, *Sol*, *TS*, *EB*, and alginate content are mostly explained by *PC*1, while variations in phenolic compounds and *OP* content are mainly explained by *PC*2. Loadings plot analysis showed that *MC*, *YM*, *TS*, and *Sol* of the films are positively correlated with their alginate content, while the samples’ *EB* is inversely correlated with their alginate content. Also, the phenolic compounds’ content shows a positive correlation with the samples’ *OP* while showing to be uncorrelated with the samples’ *MC*, *TS*, *Sol*, *EB*, and *YM*, which is in accordance with the PLS results. Therefore, samples with higher alginate concentrations can absorb more water, be more water soluble, less flexible (i.e., higher *YM*), with higher *TS* and lower *EB*. It is widely reported that gel strength depends not only on alginate and calcium concentration and polymerization degree, but also on alginate source, which confers different mannuronic and guluronic acid (M/G) ratios. These M/G ratios and M/G distribution in the alginate chains have a noticeable consequence on mechanical properties. It is considered that alginates with a high proportion of guluronic acid produce rigid gels, which contribute to the gel formation with an egg-box structure. Conversely, alginates with mainly mannuronic acids produce softer, more elastic gels [40]. A k-means cluster analysis was also conducted to group the different samples based on their similarity. By comparing the maximum distance from the cluster with the number of clusters (Figure 4A), it was possible to determine that the first deflection point occurred at a number of cluster equal to 3. Thus, it was possible to divide the PCA scores into three cluster groups: samples 1, 3, and 7 (group I); 8, 9, 10, and 11(group II); and 2, 4 and 6 (group III). In fact, these groups correspond to samples with a low, medium, and high alginate content, respectively. However, it is important to mention that the variability within the *PC*1 axis has more significance than the variability in the *PC*2 axis and, consequently, group I samples (1, 3 and 7) may be included in the group II cluster.

To further assess how the different variables correlate with each other, particularly, how the different formulations modulate *MC* and *YM* of the films and which correlations are significant, Pearson’s and Spearman’s correlation matrices were calculated (Table 6). 

It was observed that both Pearson’s and Spearman’s correlations show a significant correlation between the samples’ alginate content and their *YM*, *MC*, and *TS*. On the other hand, the amount of phenolic compounds in the samples does not show any correlation with the studied variables, which is in accordance with the PLS and PCA results.

## 3. Materials and Methods

Glycerol, alginate, ethanol, methanol, gallic acid, caffeic acid, pyrogallol, ferulic acid, chlorogenic acid, 2,2-diphenyl-1-picrylhydrazyl (DPPH), and Folin-Ciocalteu reagent (2 mol·L^−1^) were purchased from Sigma-Aldrich (Saint Louis, MI, USA).

### 3.1. Amaranth Phenolic Extraction

Amaranth (*A. cruentus*) grains were purchased from Vidal Foods S.A.C. (Lima, Peru) and stored in a cold chamber (4 °C) until further analysis. Amaranth grains were sieved, cleaned, and ground in a roller mill to obtain whole amaranth flour. Amaranth flour was stirred for 1 h at room temperature, using different extraction solvents, ethanol (80%), methanol (50%) or water, at 1:20 (amaranth:solvent) ratio. Subsequently, the organic solvents were evaporated at 35 °C under vacuum and residual extract was freeze-dried (CHRIST-Alpha 1-4 LD plus, Osterode am Harz, Germany). The dry residue weight was determined gravimetrically and stored at −20 °C until further use. 

### 3.2. Identification of Amaranth Phenolic Compounds by Ultra-High-Performance Liquid Chromatography (UHPLC)

Amaranth phenolic extracts were analyzed using the Shimatzu Nexpera X2 UHPLC (Shimadzu Corporation, Kyoto, Japan) with Diode Array Detector and SPD-M20A detector unit according to a method reported by other authors [41]. Briefly, a reverse phase Acquity UPLC BEH C18 column with 2.1 mm × 100 mm, 1.7 µm particle size (Waters, Milford, MA, USA) and a pre-column were used at 40 °C with a flow rate of 0.4 mL min^−1^. Solvent A (0.1% formic acid) and solvent B (acetonitrile) were used. The solvent B elution gradient was: 0–5.5 min, 5%; 5.5–17 min, 60%; 17.0–18.5 min, 100%, then column balance from 18.5 to 30.0 min to 5%. Phenolic compounds were identified comparing to equivalent standards. All analyses were done in triplicate.

### 3.3. Antioxidant Activity of Freeze-Dried Amaranth Extracts

Freeze-dried extract (1 g) was suspended in the 50 mL of water using a rotor-stator mixer for about 1 min, and afterwards, stirred for about 12 h at 200 rpm at room temperature. 

DPPH radical scavenging activity was determined according to other authors [42]. A DPPH stock solution (24 mg DPPH + 100 mL methanol) was prepared and subsequently, a working solution (8.6 mL stock solution + 50 mL methanol) was obtained. The reaction was performed with 150 μL of extract and 2.9 mL of working solution. After 30 min of reaction in the dark, the sample was analyzed at 515 nm. The calibration curve (25–935 μmol·L^−1^) was prepared with the water-soluble ascorbic acid (a well-known standard with strong antioxidant activity). The sample’s absorbance was related to ascorbic acid standard, and the result was calculated as ascorbic acid equivalents (ACE) in mg·g^−1^ sample.

### 3.4. Total Phenolic Content of Freeze-Dried Amaranth Extracts

Extracts’ total phenolic content was determined using Folin-Ciocalteau method. Lyophilized amaranth extract (1 g) was added to 2 mL of aqueous Na_2_CO_3_ solution (2%) in glass tubes. After 2 min, 100 mL of Folin-Ciocalteau reagent (1:1 in distilled water) was added to the mixture and vortexed. The solution was allowed to stand for 30 min at room temperature. Absorbance was measured at 750 nm against reaction blank using a spectrophotometer Beckman Coulter DU 640 (Beckman Instruments Inc., Fullerton, CA, USA). A blank sample (i.e., sample replaced by water) was prepared. Quantitative determination of total phenolic content was done on the basis of a standard curve (0.02 to 0.12 mg/mL) of gallic acid (as it is a typically used standard). Results were expressed as gallic acid equivalents (mg GAE·100 g^−1^ dry sample).

### 3.5. Experimental Design and Production of Alginate Film with Amaranth Phenolic Compounds 

A CCRD with 2 independent variables (i.e., 2^2^), alginate (0.75–1.50% *w*/*v*) and phenolic compounds (0.0–2.0% *w*/*v*) concentration; 3 replicates at the central point (assays 9, 10, and 11); and 4 axial points, giving a total of 11 assays, was used for the design and optimization of alginate films with amaranth phenolic compounds. Once the final alginate-glycerol mixture concentration was fixed at 1.5%, the glycerol concentration was calculated based on the alginate content. The alginate-glycerol mixture concentration was chosen based on a previous study [43]. The different films’ formulations are described in Table 7.

Alginate film-forming solutions were prepared by dissolving alginate in stirring distilled water (350 rpm) for 30 min at 20 °C. Then, glycerol was added and solutions were stirred (350 rpm) for 30 min at 20 °C until a homogeneous solution was obtained. The freeze-dried phenolic compounds were added to solutions according to concentrations defined in Table 7. The solutions were heated at 40 °C and stirred for 1 h at 900 rpm. The solutions were transferred (28 mL) to Petri dishes and dried in an oven at 35 °C for 20 h. After drying, the films were stored at 20 ± 2 °C at 53% of relative humidity in desiccators containing Mg(NO_3_)_2_·6H_2_O-saturated solution until further investigation.

### 3.6. Characterization of the Alginate-Based Films

#### 3.6.1. Scanning Electron Microscopy (SEM)

Films’ surface morphology was analyzed by SEM (Phenom-World BV, Eindhoven, The Netherlands). All results were acquired using the ProSuite software. Samples 1, 5, 7, and 9 were added to aluminum pin stubs with electrically conductive carbon adhesive tape (PELCO Tabs™). Samples were coated with 2 nm of Au (20 Å) for improved conductivity. The aluminum pin stub was then placed inside a Phenom Standard Sample Holder and analyzed with surface topography at 5 kV. Various film surface areas (10 × 10 mm) were checked using touch mode.

#### 3.6.2. Water Contact Angle

The sessile drop method was performed to measure water contact angle. Five microliters of a water droplet was placed on the film surface using an automatic piston syringe (Hamilton, Biel, Switzerland) and the water contact angle measurements (made in triplicate) were performed after 180 s using an optical contact angle meter (OCA 20, Dataphysics, Filderstadt, Germany).

#### 3.6.3. Film Solubility (*Sol*)

Films’ solubility in water was performed using the method reported by other authors [44]. Briefly, film discs (diameter = 2 cm) were weighed (*m_i_*) and placed in a cup containing 50 mL of water at 20 °C. After 24 h, the film samples were collected and placed in an oven (105 °C) until constant weight (*m_f_*) in order to establish dry sample weight that was not solubilized. The solubility (%) of the films in water was determined as follows:

(1) $$\mathrm{Solubility}=\frac{{(m}_{i}{-\;m}_{f})\;}{m_{i}}\;\times \;100$$

where *m_i_* is the initial sample mass, and *m_f_* is the final sample mass.

#### 3.6.4. Moisture Content (*MC*)

Moisture content (*MC*) was determined according to other authors [27]. Briefly, film samples (20 mg) were dried at 105 °C in an oven for 24 h to determine the water content removed from the film sample. The test was conducted in triplicate on each film sample.

#### 3.6.5. Water Vapor Permeability (*WVP*)

*WVP* was assessed using ASTM E96-92 method [27]. Three samples (diameter = 5.1 cm) were cut from each film sample, sealed on a permeation cell cup (containing distilled water at 100% RH; 2.337 × 10^3^ Pa vapor pressure at 20 °C), and placed in a desiccator containing silica gel (0 % RH; 20 °C). The water transferred through the films was determined from cell cup weight loss over time. The cups were weighed until steady state was achieved (approximately 10 h) to determine weight loss. Weight loss of the cell cups was plotted as a function of time and the slope of the linear regression line of each sample was calculated (r^2^ > 0.9), and the water vapor transmission rate (*WVTR*) was calculated from the slope of the linear regression (g s^−1^) divided by the film area (m^2^). For each type of film, *WVP* measurements were replicated three times. *WVP* (g m^−1^ s^−1^ Pa^−1^) was calculated using Equation (2):

(2) $$\mathrm{WVP}=\frac{\left(\mathrm{WVTR}\;\times \;L\right)}{\Delta P}$$

where *WVTR* is the water vapor transmission rate (g m^−2^ s^−1^), *L* is the mean film thickness (m), and Δ*P* is the partial water vapor pressure difference (Pa) across the film. 

#### 3.6.6. Thermal Analysis

Thermal profiles of all films were evaluated by thermogravimetric analysis (TGA) as described by other authors [44]. Film samples (approximately 5 mg) were placed under high pressure in stainless steel pans. Film samples were heated from 20 to 580 °C, at 10 °C.min^−1^, under nitrogen atmosphere, in triplicate, using a TGA 4000 equipment (Perkin Elmer, Waltham, MA, USA).

Differential scanning calorimetry (DSC) measurements were performed using a DSC 6000 equipment (Perkin Elmer, Waltham, MA, USA). A film sample (10 mg) was placed in aluminum pans and measurements were taken from 20 to 250 °C at 10 °C min^−1^ on nitrogen atmosphere. DSC data was analyzed and melting enthalpy change (Δ*H_m_*) and melting temperature peak (*T_m_*) were determined.

#### 3.6.7. Mechanical Properties

Tensile strength (*TS*), elongation at break (*EB*), and Young’s modulus (*YM*) of the film samples were measured in a Texture Analyzer TA-XT2i (Stable Microsystems, Surrey, UK) according to ASTM D 638-99 standard method [45]. The samples (45 × 20 mm^2^ strips) were fixed between the grips with an initial 100 mm separation, and the cross-head speed was set at 0.8 mm s^−1^. At least five measurements were completed for each formulation.

#### 3.6.8. Film Thickness

Film thickness was assessed using a digital micrometer device no. 293-5 (Mitutoyo, Sakado, Takatsu-ku, Kawasaki, Kanagawa, Japan) with a 0.001 mm resolution. Ten measurements were randomly performed on each film sample.

#### 3.6.9. Optical Properties (OP)

Film optical properties were evaluated [44]. Briefly, films’ brightness (L*), a*, and b* parameters were measured. Film samples were placed on top of a white standard plate (Y = 93.5, x = 0.3114, y = 0.3190), and measurements were performed using a colorimeter (Minolta CR 300, Tokyo, Japan). All samples were evaluated in triplicate. Film samples’ opacity (*OP*) was performed using the Hunterlab method (i.e., the ratio between CIE tristimulus Y value on a black standard and the CIE tristimulus Y value on a white standard). Six measurements were taken of each sample, and three samples of each film were tested.

### 3.7. Statistical Analyses

Statistical analyses were performed using Excel 2019 software (Microsoft Windows, Microsoft Corporation, Redmond, WA, USA) and SPSS 22.0 software (IBM, Chicago, IL, USA). One-way analysis of variance (ANOVA) with Tukey’s multiple comparisons test was performed to determine the significance of differences with α = 0.05. The CCRD experimental design tables and results were obtained in Protimiza Experiment Design Software (Protimiza, Campinas, Brazil).

Principal component analysis (PCA); Partial Least Squares (PLS) using the variable importance of projections (VIP) score to determine the variable importance where a VIP above 0.8 indicates that the independent variable significantly explains the variability of the dependent variables [46]; the k-means cluster analysis (the number of clusters was determined using the elbow rule) [47]; the box plot (for outlier detection); and Pearson’s and Spearman’s correlation matrices, with a 2-tailed test of significance with α = 0.05 to obtain the significance level of the correlation coefficients, were obtained using Origin Pro 2018 software (Origin-Lab Corporation, Northampton, MA, USA). All film samples were analyzed except sample 6 because it was not possible to handle the film sample, and consequently, it was not possible to characterize it.

## 4. Conclusions

This study demonstrated that the amaranth phenolic compounds extraction using ethanol is adequate to obtain a product with high phenolic compounds retention and antioxidant capacity. Moreover, amaranth phenolic compounds were successfully incorporated in films composed of alginate and glycerol. The results of this work provided useful information on the structural properties of alginate-based films incorporating amaranth phenolic compounds and on the structural changes that occur in the film network induced by mixing different components proportions. Furthermore, this study can be used to tailor alginate films containing amaranth phenolic compounds, depending on their final food application. If higher mechanical stability is a priority, films presenting high alginate concentration should be produced. On the other hand, if the film’s flexibility is prioritized, the alginate concentration on the films should be decreased and consequently, glycerol concentration increased. Also, the phenolic compounds concentration in the film samples did not significantly influence the studied response variables.

In conclusion, amaranth phenolic compounds incorporation into alginate films is an excellent option for developing edible and environmentally friendly packaging materials to apply to a broad number of food products. Further studies are required to evaluate films’ antioxidant capability in food products during storage.

## Figures and Tables

**Table 1:** Total phenolic content and antioxidant activity (AA) of *Amaranthus cruentus* sp. extracts in different solvents and after freeze-drying.

Solvent	TPC (mg GAE/g)	AA (mg ACE/g)
Methanol (50%) ^1^	3.9 ± 0.12 ^a^	0.24 ± 0.01 ^a^
Ethanol (80%) ^1^	3.5 ± 0.09 ^a^	0.21 ± 0.01 ^b^
Water	2.4 ± 0.07 ^b^	0.12 ± 0.02 ^b^
Freeze-dried ethanol extract	16.78 ± 2.61 ^c^	7.54 ± 0.29 ^c^

Abbreviations: GAE gallic acid equivalent; ACE ascorbic acid equivalent; TPC total phenolic content. ^1^ Expressed as a solvent fraction in water. ^a–c^ Different letters in the same column indicate statistical differences between samples (*p* < 0.05).

**Table 2:** Phenolic acids and flavonoids present in the freeze-dried amaranth (*Amarathus cruentus* sp.) grain extract.

Phenolic Compounds	Concentration (mg/L)
Vanillic acid	6.08 ± 0.01
Ferulic acid	1.96 ± 0.02
*p*-coumaric acid + epicatechin	1.66 ± 0.00
*o*-coumaric acid	1.32 ± 0.00
Ellagic acid	1.08 ± 0.13
Cinnamic acid	2.23 ± 0.01
Resveratrol	4.07 ± 0.02
Rutin	2.42 ± 0.00
Total	20.82 ± 0.10

**Table 3:** Water contact angle (180 s after the water drop deposition), moisture content (*MC*), solubility (*Sol*), water vapor permeability (*WVP*), enthalpy of melting (Δ*H_m_*), glass transition temperature (*T_g_*), onset temperature (*T_onset_*), and TGA peak (*TGA_p_*) for alginate films with amaranth phenolic compounds.

Films	Water Contact Angle (°)	*MC* (%)	*Sol* (%)	*WVP* × 10^10^ [g·(Pa·s·m)^−1^]	Δ*H_m_* (J g^−1^)	*T_g_* (°C)	*T_onset_* (°C)	*TGA_p_* (°C)
1	35.30 ± 0.75 ^b,c^	47.76 ± 1.22 ^b^	85.63 ± 2.54 ^b^	0.28 ± 0.02 ^a^	471.15 ± 11.63 ^a^	148.56 ± 3.66 ^a^	188.63 ± 0.04 ^b,c^	214.47 ± 0.80 ^b^
2	0.00 ± 0.00 ^a^	72.06 ± 0.52 ^a^	93.88 ± 1.21 ^a^	0.26 ± 0.03 ^a^	423.94 ± 90.07 ^a.b^	144.85 ± 2.38 ^a^	209.55 ± 0.06 ^a^	218.03 ± 0.16 ^a,d^
3	47.10 ± 1.28 ^b^	49.60 ± 1.18 ^b^	79.67 ± 1.55 ^c^	5.43 ± 0.22 ^c^	213.48 ± 19.62 ^c^	123.68 ± 1.07 ^b^	192.51 ± 2.25 ^b^	213.47 ± 0.57 ^b^
4	0.00 ± 0.00 ^a^	72.13 ± 0.68 ^a^	98.86 ± 1.98 ^a^	3.70 ± 0.41 ^b^	290.51 ± 5.48 ^c^	125.23 ± 3.83 ^b^	209.21 ± 3.40 ^a^	219.07 ± 1.76 ^a^
5	21.30 ± 3.94 ^c^	35.75 ± 0.85 ^c^	87.68 ± 1.38 ^b^	5.63 ± 0.28 ^c^	504.97 ± 38.19 ^a^	153.94 ± 3.91 ^a^	184.96 ± 3.27 ^b,c^	213.78 ± 0.57 ^b^
6	27.97 ± 0.67 ^c^	74.68 ± 0.55 ^a^	97.74 ± 0.82 ^a^	5.15 ± 0.32 ^c^	294.81 ± 51.19 ^c^	126.00 ± 3.03 ^b^	181.89 ± 3.48 ^c^	209.69 ± 0.11 ^c^
7	29.33 ± 1.55 ^b,c^	53.57 ± 0.82 ^d^	97.78 ± 0.71 ^a^	3.08 ± 0.15 ^d^	532.61 ± 19.23 ^a^	153.31 ± 1.08 ^a^	193.71 ± 1.94 ^b^	215.79 ± 0.08 ^b,d^
8	21.87 ± 18.94 ^c^	57.57 ± 0.91 ^e^	79.20 ± 1.24 ^c^	2.72 ± 0.32 ^d^	230.73 ± 30.06 ^c^	124.54 ± 1.07 ^b^	193.04 ± 3.13 ^b^	216.08 ± 1.17 ^a,b^
9	0.00 ± 0.00 ^a^	63.99 ± 1.87 ^f^	74.91 ± 1.95 ^d^	2.66 ± 0.18 ^d^	469.75 ± 9.57 ^a,b^	148.23 ± 6.65 ^a^	187.17 ± 0.18 ^b,c^	215.20 ± 0.24 ^b,d^
10	0.00 ± 0.00 ^a^	55.04 ± 1.00 ^d^	66.20 ± 1.20 ^e^	2.44 ± 0.12 ^d^	425.27 ± 42.19 ^a^^,b^	149.59 ± 9.91 ^a^	193.48 ± 0.69 ^b^	214.71 ± 0.78 ^b^
11	0.00 ± 0.00 ^a^	67.03 ± 1.52 ^f^	60.06 ± 1.50 ^f^	2.57 ± 0.19 ^d^	413.16 ± 57.00 ^a,b^	155.64 ± 2.62 ^a^	191.04 ± 3.92 ^b^	215.14 ± 0.05 ^b,d^

^a–f^ Different letters in the same column indicate statistical differences between samples (*p* < 0.05).

**Table 4:** Thickness, color parameters (L*, a*, and b*), and opacity (*OP*) of alginate films incorporating amaranth phenolic compounds.

Films	Thickness (mm)	*OP* (%)	Color Parameters		
			L*	a*	b*
1	5.00 ± 0.26 ^a^	7.80 ± 0.53 ^b^	94.26 ± 0.28 ^b^	−0.21 ± 0.08 ^b^	6.98 ± 0.52 ^b,d^
2	5.03 ± 1.01 ^a^	9.86 ± 0.87 ^a^	95.26 ± 0.31 ^a^	0.00 ± 0.02 ^a,c^	4.08 ± 0.21 ^a^
3	6.43 ± 0.65 ^a^	7.60 ± 1.93 ^b^	94.13 ± 0.34 ^b^	−0.22 ± 0.12 ^b^	7.56 ± 0.49 ^b^
4	6.57 ± 0.32 ^a^	10.44 ± 0.48 ^a^	95.10 ± 0.16 ^a^	−0.06 ± 0.09 ^a,c^	4.76 ± 0.30 ^a^
5	5.00 ± 0.75 ^a^	3.35 ± 0.29 ^c^	96.49 ± 0.07 ^c^	−0.09 ± 0.02 ^a,b^	2.93 ± 0.23 ^c^
6	6.10 ± 1.00 ^a^	10.11 ± 0.73 ^a^	95.22 ± 0.36 ^a^	0.03 ± 0.02 ^c^	4.25 ± 0.23 ^a^
7	5.47 ± 0.86 ^a^	5.31 ± 0.42 ^d^	93.97 ± 0.29 ^b^	−0.20 ± 0.06 ^b^	6.62 ± 0.41 ^d,e^
8	4.83 ± 0.63 ^a^	9.14 ± 0.58 ^a,b^	94.51 ± 0.47 ^b^	−0.07 ± 0.07 ^a,c^	6.06 ± 0.91 ^e^
9	4.73 ± 0.85 ^a^	12.09 ± 0.51 ^e^	95.44 ± 0.23 ^b^	−0.20 ± 0.05 ^b^	7.77 ± 0.27 ^b^
10	4.33 ± 0.35 ^a^	12.00 ± 0.21 ^e^	94.59 ± 0.18 ^b^	−0.29 ± 0.02 ^b^	7.35 ± 0.48 ^b^
11	4.03 ± 0.35 ^a^	12.02 ± 0.60 ^e^	94.02 ± 0.63 ^b^	−0.30 ± 0.07 ^b^	8.33 ± 0.56 ^b^

^a–e^ Different letters in the same column indicate statistical differences between samples (*p* < 0.05).

**Table 5:** Central Composite Rotatable Design (CCRD) results (*F_calc_* and *F_tab_* represent the calculated and tabulated *F* distribution at a 5% significance, respectively).

Response Variables	Model	r^2^	*p-Value*	*F_calc_*	*F_tab_*
*Sol*	$\mathrm{Sol}=67.06+12.55{\;\mathrm{Al}}^{2}+10.44{\;\mathrm{PC}}^{2}$	0.6785	0.01069	8.4	4.46
*MC*	MC=59.02+12.74 Al	0.8763	0.00002	63.8	5.12
*WVP*	NA	NA	0.48726	1.0	5.05
*CA*	NA	NA	0.31137	1.6	5.05
*T_g_*	NA	NA	0.76410	0.5	5.05
*YM*	$\mathrm{YM}=0.31+4.32\;\mathrm{Al}+3.35{\;\mathrm{Al}}^{2}$	0.9685	0.00001	122.9	4.46
*OP*	$\mathrm{OP}=12.04+1.41\;\mathrm{Al}\;-2.17{\mathrm{Al}}^{2}\;-1.92{\mathrm{PC}}^{2}$	0.7789	0.01077	8.2	4.35
*TGA_p_*	NA	NA	0.76415	0.5	5.05
*TS*	$\mathrm{TS}=1+5.90\;\mathrm{Al}+4.50{\;\mathrm{Al}}^{2}$	0.8852	0.00017	30.9	4.46
*EB*	$\mathrm{EB}=12.92\;-6.89{\mathrm{Al}}^{2}$	0.7859	0.00028	33.0	5.12

Abbreviations: *Al*, alginate; *PC*, phenolic compounds; *Sol*, solubility; *MC*, moisture content; *WVP*, water vapor permeability; *CA*, contact angle; *T_g_*, glass transition temperature; *YM*, Young Modulus; *OP*, opacity; *TGAp*, TGA peak; *TS*, tensile strength; *EB*, elongation at break; NA, not applicable.

**Table 6:** Pearson’s and Spearman’s correlation matrices.

**Pearson Correlations**								
	***Al***	***PC***	***Sol***	***MC***	***YM***	***OP***	***TS***	***EB***
***Al***	1.00	0.00	0.48	0.92	0.86	0.35	0.81	−0.44
***PC***	0.00	1.00	−0.23	0.09	−0.08	0.31	−0.06	−0.10
***Sol***	0.48	−0.23	1.00	0.28	0.63	−0.55	0.52	−0.75
***MC***	0.92	0.09	0.28	1.00	0.79	0.54	0.72	−0.40
***YM***	0.86	−0.08	0.63	0.79	1.00	0.14	0.94	−0.77
***OP***	0.35	0.31	−0.55	0.54	0.14	1.00	0.18	0.27
***TS***	0.81	−0.06	0.52	0.72	0.94	0.18	1.00	−0.60
***EB***	−0.44	−0.10	−0.75	−0.40	−0.77	0.27	−0.60	1.00
**Spearman Correlations**								
	***Al***	***PC***	***Sol***	***MC***	***YM***	***OP***	***TS***	***EB***
***Al***	1.00	0.00	0.44	0.93	0.93	0.35	0.93	−0.45
***PC***	0.00	1.00	−0.21	0.19	−0.15	0.21	−0.13	−0.04
***Sol***	0.44	−0.21	1.00	0.24	0.38	−0.50	0.27	−0.71
***MC***	0.93	0.19	0.24	1.00	0.85	0.55	0.87	−0.40
***YM***	0.93	−0.15	0.38	0.85	1.00	0.45	0.97	−0.33
***OP***	0.35	0.21	−0.50	0.55	0.45	1.00	0.53	0.38
***TS***	0.93	−0.13	0.27	0.87	0.97	0.53	1.00	−0.30
***EB***	−0.45	−0.04	−0.71	−0.40	−0.33	0.38	−0.30	1.00

Values marked as red correspond to significant correlation between the dependent and independent variables (*p* < 0.05). Abbreviations: *Al*, alginate; *PC*, phenolic compounds; *Sol*, solubility; *MC*, moisture content; *YM*, Young Modulus; *OP*, opacity; *TS*, tensile strength; *EB*, elongation at break.

**Table 7:** Composition of the different film formulations produced with alginate (alginate + glycerol content fixed at 1.5 % (*w*/*v*) and glycerol values are presented in parentheses) and amaranth phenolic compounds using a 2^2^ central composite rotatable design (CCRD).

Formulation	Alginate (% *w*/*v*)	Phenolic Compounds (% *w*/*v*)
1	0.86 (0.64)	0.30
2	1.39 (0.11)	0.30
3	0.86 (0.64)	1.70
4	1.39 (0.11)	1.70
5	0.75 (0.75)	1.00
6	1.50 (0.00)	1.00
7	1.13 (0.37)	0.00
8	1.13 (0.37)	2.00
9	1.13 (0.37)	1.00
10	1.13 (0.37)	1.00
11	1.13 (0.37)	1.00

## Data Availability

Not applicable.
